# AI in microbiome‐related healthcare

**DOI:** 10.1111/1751-7915.70027

**Published:** 2024-11-02

**Authors:** Niklas Probul, Zihua Huang, Christina Caroline Saak, Jan Baumbach, Markus List

**Affiliations:** ^1^ Institute for Computational Systems Biology University of Hamburg Hamburg Germany; ^2^ Data Science in Systems Biology, TUM School of Life Sciences Technical University of Munich Freising Germany; ^3^ Computational Biomedicine Lab, Department of Mathematics and Computer Science University of Southern Denmark Odense Denmark; ^4^ Munich Data Science Institute Technical University of Munich Garching Germany

## Abstract

Artificial intelligence (AI) has the potential to transform clinical practice and healthcare. Following impressive advancements in fields such as computer vision and medical imaging, AI is poised to drive changes in microbiome‐based healthcare while facing challenges specific to the field. This review describes the state‐of‐the‐art use of AI in microbiome‐related healthcare. It points out limitations across topics such as data handling, AI modelling and safeguarding patient privacy. Furthermore, we indicate how these current shortcomings could be overcome in the future and discuss the influence and opportunities of increasingly complex data on microbiome‐based healthcare.

## ARTIFICIAL INTELLIGENCE IN MICROBIOME RESEARCH

In recent years, it has become increasingly evident that human health is tightly linked to the human microbiome (Pruss & Sonnenburg, 2021; Rao et al., 2021; Talmor‐Barkan et al., 2022). Conversely, non‐communicable human diseases such as inflammatory bowel disease are related to an imbalance in the gut microbiome, often called dysbiosis (Santana et al., 2022; Yu, 2018). The term dysbiosis, which is widely used, is ill‐defined and disputed (Brüssow, 2020). It relates to the general concept that changes in microbial composition are associated with a disease. These changes are most evident when a significant loss of microbiota richness occurs, but many studies now aim at identifying more subtle disease‐associated microbial signatures. Generally, microbiome‐related healthcare aims to identify changes in microbial profiles that can be used to diagnose a disease and that can be used to suggest therapies for restoring a healthy microbiota. Countless companies have recognized this potential and offer microbiome tests along with probiotics and food supplements to treat a microbiome imbalance. However, a recent study has shown that the claims of these companies are mostly not substantiated by research and lack clinical validation (Hoffmann et al., 2024). This is unsurprising, as the complexity of microbial communities in general and host–microbiome interactions in particular is far from understood (Moreno‐Indias et al., 2021). With the increasing availability of heterogeneous and complex microbiome‐related data, however, integrating artificial intelligence (AI) into microbiome research has become increasingly popular and necessary (Hernández Medina et al., 2022) as it offers a promising avenue for advancing microbiome‐related healthcare as presented in this review. Various and partially conflicting definitions of AI exist, but we will attempt to clarify what we mean in this context. When researchers speak of AI, they typically focus on creating systems capable of performing tasks that require a certain amount of intelligence, like pattern recognition, decision‐making or natural language processing. AI tools go beyond statistical learning methods, which can also recognize patterns but need more explicit programming to fulfil a task. Researchers also often speak of classical machine learning when they refer to simpler models that perform adequately on smaller datasets. In contrast, contemporary AI methods such as deep learning are much more complex but require vastly more data and computational power. While AI has the potential to be applied to nearly all parts of microbiome analysis, we only highlight an excerpt of available AI methods and applications in microbiome‐related healthcare as an introduction to the field.

AI has already been instrumental in advancing microbiome data analysis, for instance, by increasing the quality of microbial genomes constructed from patient samples and improving the detection of novel microbes, genes and metabolic pathways (Sun et al., 2023). It is also frequently used in preprocessing pipelines for microbiome‐related analysis tasks such as taxonomic annotation (Bokulich et al., 2018) and feature selection (Peng et al., 2005; Queen & Emrich, 2021).

Furthermore, AI has advanced biomarker detection for drug discovery (Bohr & Memarzadeh, 2020), pathogen classification and detection (Jiang et al., 2022) as well as predicting disease susceptibility, progression and treatment response (Routy et al., 2018) based on microbial compositions found in, for example, the gut microbiome (Giuffrè et al., 2023). For example, Gacese et al. created random forest and logistic regression prediction models based on patient's gut microbiome metagenomics data, faecal calprotectin (FCal), human beta defensin 2 (HBD2) and chromogranin A (CgA) to distinguish Inflammatory bowel disease (IBD) and irritable bowel syndrome (IBS) in a non‐invasive way, which aim to reduce the number of endoscopies needed (Gacesa et al., 2021). This holds for communicable diseases, but also for non‐communicable diseases whose origin and onset are harder to classify and detect. For instance, the role of the microbiome in autoimmune diseases such as graft‐versus‐host disease, inflammatory bowel disease and Type 2 diabetes is recognized but not clearly understood, limiting the clinical application of microbiome‐associated diagnostics and therapy.

The highly individual composition of the human microbiome is a strong argument for developing personalized medicine approaches and personalized nutrition (Ratiner et al., 2023). For example, Zeevi et al. built a model using relative abundances of 16S rRNA‐based phyla and other features (e.g. meal features, clinical features and dietary habits) to predict the personalized postprandial glycemic response to real‐life meals (Zeevi et al., 2015). Towards this goal, AI can improve the cultivation of microbial communities (Li, Liu, et al., 2023; Selma‐Royo et al., 2023) and, in turn, help to model and subsequently develop synthetic communities with desired properties and behaviours (Mabwi et al., 2021). This could also help transition from donor‐based faecal transplants to more flexible synthetic community‐based therapies (van Leeuwen et al., 2023).

Although AI clearly shows the capabilities to revolutionize the field of microbiome‐related healthcare, we will highlight the various challenges that need to be addressed before it can be applied effectively. The decision‐making process of AI models needs to be well understood through concepts such as ‘explainable AI’ to generate actionable insights. Further, we need more heterogeneous and diverse datasets to create models that generalize well (i.e. perform well on unseen data). While data quality rises, we need to establish models that perform under challenging conditions, such as with noisy or lower quality data.

Compared to AI‐driven advances in other healthcare fields, such as medical imaging and radiomics, microbiome‐related healthcare arguably lags behind (McCoubrey et al., 2021). Contributing factors include, but are not limited to, insufficient high‐quality data to cope with the sizable inter‐individual and geographic heterogeneity already observed in the healthy human microbiome (Olsson et al., 2022), often unclear functional annotations and a lack of standardized cooperation between institutions. More specifically, we are lacking robust datasets with a large number of samples. Because microbiomes are highly diverse across individuals and populations, it is difficult to distinguish between the true signal and batch effects caused by, for example, different primers in 16S sequencing or different DNA extraction protocols. In general, institutes will have to collaborate to gather more data. However, due to legal barriers and privacy concerns, this is challenging.

This review covers the most important state‐of‐the‐art microbiome analysis approaches in applied healthcare and research and raises awareness for emerging challenges. We consider the types of microbiome‐related data available for AI development and application, assess existing and emerging use cases for AI in microbiome‐related healthcare and discuss current roadblocks and future perspectives.

## COMPREHENSIVE MOLECULAR PROFILING IN MICROBIOME RESEARCH

Data are the foundation of all AI research. Thus, it is important to understand the properties and applications of different omics data in the field of microbiome‐related healthcare.

**FIGURE 1:**
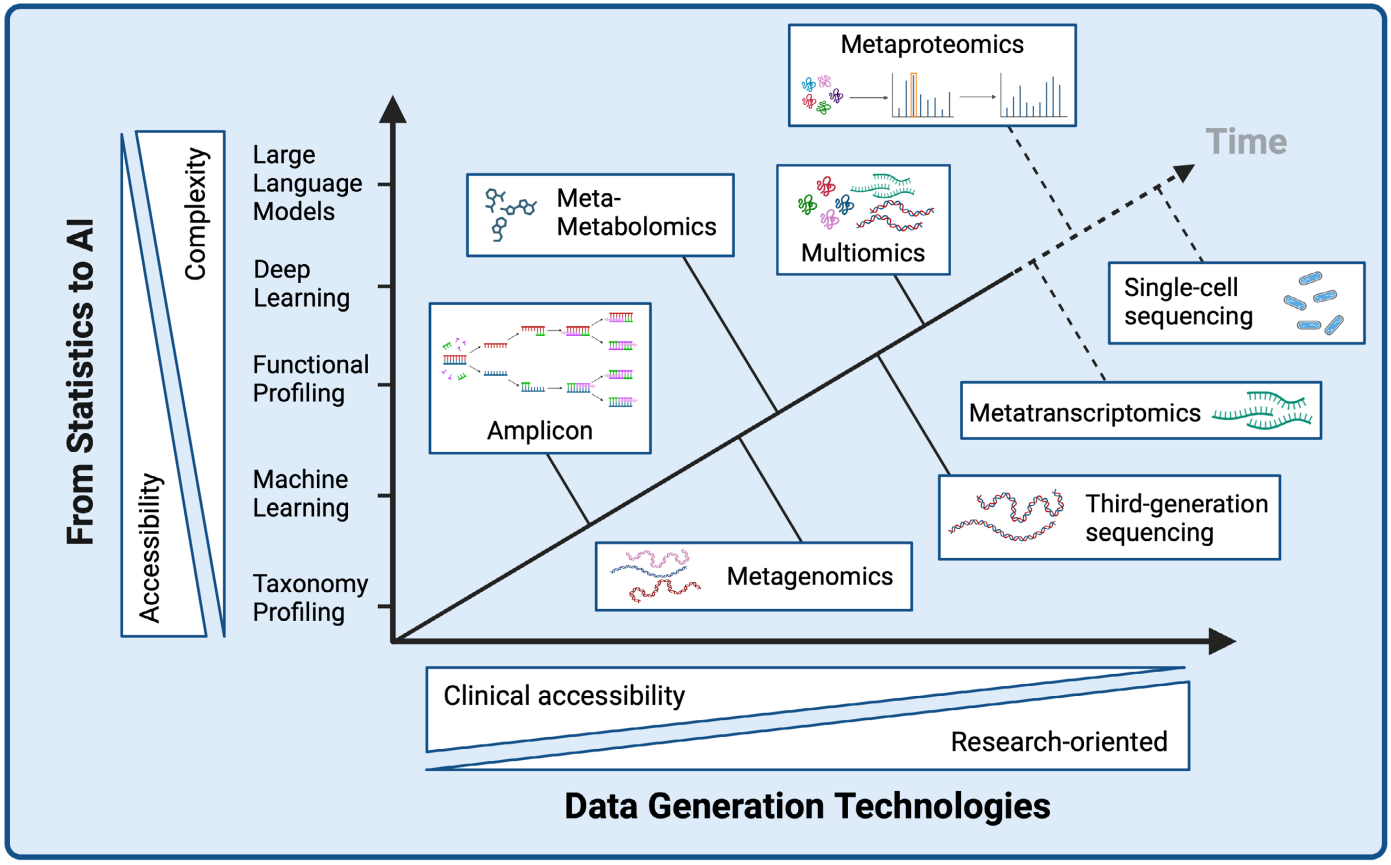
Current and emerging landscape of applying AI in microbiome‐related healthcare. Created with BioRender.com.

Several technologies are used for molecular microbiome profiling across different omics levels. Differences in accessibility (e.g. costs, scalability and turnaround time) and complexity (e.g. type and volume of data) affect whether these technologies are used rather for research‐oriented or clinical applications (Figure 1). The most prevalent and cost‐effective method for microbiome taxonomy profiling is amplicon sequencing of the 16S rRNA gene, which allows studying microbiome composition and richness but offers limited insights into functional characteristics related to human health (Matchado et al., 2023). 16S rRNA gene sequencing is also subject to considerable biases (Abellan‐Schneyder et al., 2021) and has a low taxonomic resolution (up to the genus level). While full‐length 16S intragenomic copy variants can potentially provide taxonomic resolution at species and strain levels (Johnson et al., 2019), microbial research is shifting towards metagenomic sequencing (MGS), which offers not only strain‐level resolution but also vastly improved functional annotation (Yen & Johnson, 2021). The advantages of short‐read sequencing technologies such as Illumina (higher quality reads) and those of third‐generation long‐read sequencing platforms, such as Oxford Nanopore and Pacific Biosciences (longer reads), can be jointly leveraged to recover higher quality metagenome‐assembled genomes, for example, producing assemblies with fewer contigs, higher total number of assembled sequences and significantly higher N50 values (Chen et al., 2022).

In principle, metatranscriptomics could offer a more representative view of the functional activity as compared to metagenomics. However, metatranscriptomics is currently more challenging compared to metagenomics because of the short half‐life of prokaryotic mRNA and difficulty in bioinformatics analysis (e.g. assigning reads to specific species/strain) (Ojala et al., 2023). Metabolomics complements this further by quantifying metabolites driving the interactions within and between the microbiota. Targeted metabolomics excel in metabolite annotation and interpretation, but this requires a priori knowledge of the molecules of interest before measurement (Alarcon‐Barrera et al., 2022). Untargeted metabolomics aims to measure as many metabolites as possible in a sample, but its effective use suffers from a lack of consensus about methods and difficulties in robustly annotating unknown metabolites in the absence of well‐established standards (Roach et al., 2024). The emerging field of metaproteomics could bridge the gap between metatranscriptomics and metabolomics and offer insights into signalling peptides, which play a crucial role in host‐microbiome interaction (Cheng et al., 2019). However, metaproteomics applications remain limited due to challenges such as false‐positive peptide matches during database construction (Miura & Okuda, 2023).

An untapped potential lies in integrating information across omics layers (Jiang et al., 2019; Sankaran & Holmes, 2019). As microbiome‐related diseases can affect different functional layers, combining multiple omics types is essential in understanding the full impact on the disease host (Muller et al., 2024). This has already been explored successfully in pilot studies, such as a study that linked specific bacteria proteases with disease severity in ulcerative colitis (Mills et al., 2022). Notably, this is also still an open research challenge in other fields, such as oncology, where initiatives such as the Cancer Genome Atlas have made huge multi‐omics datasets available more than a decade ago (Agamah et al., 2022; Cancer Genome Atlas Research Network et al., 2013). Further untapped potential lies in the incorporation of medical record data that capture the patient's full medical history and contain information about a donor's lifestyle and diet to account for the dynamic nature of the microbiome as it can be affected by various environmental factors (e.g. disease state, diet and age) (Dong & Gupta, 2019; Spor et al., 2011).

In contrast to the bulk profiling approaches introduced thus far, single‐cell sequencing has the potential to disentangle the complex interactions and unique contributions of taxa within microbiota. Single‐cell protocols such as DoTA‐seq, SMH‐seq and Microbe‐seq (Lan et al., 2024; Lötstedt et al., 2023; Zheng et al., 2022) have been developed to address unique challenges due to considerable variance in the size of microbial species, low amounts of genetic material and high risk of premature lysis or permeabilization due to the properties of microbial cell membranes. Additional methodological approaches help to chart horizontal gene transfer of mobile genetic elements (MGEs) as a driving force in the genetic composition and diversity of environments. For instance, combining MGS and MetaHi‐C technology allows assigning MGEs to their host bacteria (Marbouty et al., 2021) which can aid in the tracking of MGEs to predict the spread of antibiotic resistance genes within microbiomes (Ellabaan et al., 2021).

While the most suitable profiling method for any given application has to be determined on a case‐by‐case basis, there is a clear trend towards creating more complex data through technologies that allow deeper or more high‐throughput profiling or the combination of multiple datasets into one.

## APPLYING AI TO MICROBIOME‐RELATED DATA

Specifically, the application of AI to microbiome data analysis has to contend with unique challenges, such as its compositional nature, high dimensionality, technical variability, missing data and integration needs (Papoutsoglou et al., 2023). Like most molecular data, microbiome data suffers from the curse of dimensionality, where many features are highly correlated (D'Elia et al., 2023). The composition of communities in microbial data is also highly sparse and heterogeneous, precluding its use in methods that assume a specific distribution of the input data.

Nonetheless, AI has already been frequently applied to microbiome‐related data for taxonomic and functional annotation, phenotype prediction, biomarker discovery, patient stratification and classification of disease subtypes (Beghini et al., 2021; Ghaemi et al., 2019; Li, Wang, et al., 2023; Liu et al., 2022; Su et al., 2022; Thomas et al., 2019). For example, an oral and faecal microbiota‐based random forest (RF) classifier has been developed to distinguish individuals with colorectal cancer (CRC) from healthy individuals using log‐transformed operational taxonomic unit (OTU) data (Flemer et al., 2018). In another example, 33 gene biomarkers were used to identify individuals with CRC based on MGS sequencing (Gupta et al., 2019). However, most studies reporting successful classifiers did not include independent validation data, such that the potential for generalization and clinical applicability often remains questionable. For instance, a random forest classifier trained for predicting Type 2 diabetes (Reitmeier et al., 2020) initially showed promising results but failed when applied to an independent cohort from a different geographic location. This emphasizes the need for cross‐regional studies, data integration and more complex methods that can counteract such biases or be retrained for local use.

While classical machine learningI methods are mostly applied to classification tasks in the microbiome field, contemporary methods like deep learning (DL) methods involving various artificial neural network architectures often demonstrate superior performance in identifying complex and non‐linear patterns (e.g. low‐dimensional representation of features), but require more training data. For example, EnsDeepDP learns a low‐dimensional representation of the microbiome data as input for a classical machine learning‐based classification model and performs better than existing algorithms (Shen et al., 2023). Alternatively, methods such as Met2Img, DeepMicro, metaNN and megaD combine convolutional neural networks (CNN) and autoencoders for data compression and classification (Lo & Marculescu, 2019; Mreyoud et al., 2022; Nguyen et al., 2018; Oh & Zhang, 2020). Graph and network analysis have a long tradition in microbial research, particularly modelling interactions between different taxa (Matchado et al., 2021). More recently, graph neural networks (GNN) have allowed combining the advantages of graph structures with those of neural networks. For example, GNNs have been used to construct disease prediction models from gut metagenomics data (Syama et al., 2023). Longitudinal data are still relatively rare in spite of its ability to capture dynamic changes in the microbiome. However, methods such as phyLoSTM, a combination of CNN and long short‐term memory networks (LSTM), can leverage time‐course data to associate changes in the microbiota with the host's environmental factors, leading to improved disease prediction (Sharma & Xu, 2021). Moreover, methods such as DeepARG aim to identify antibiotic resistance genes (ARGs) and antimicrobial peptides (Arango‐Argoty et al., 2018). While the application of AI in metagenomics data and gene discovery is impressive, most microbial genes only have rudimentary or no functional annotation. Although AI has previously been used for creating reference‐based functional annotations through the application of probabilistic ML methods like hidden Markov models (Finn et al., 2011), DL‐based functional annotation pipelines surpass reference‐based annotation, allowing for the discovery of new functional groups and patterns (Maranga et al., 2023; Pavlopoulos et al., 2023). Also, for emerging single‐cell profiling data, suitable DL‐based tools have been proposed, including ScAnCluster, scDFC and scDeepCluster, which are better suited to clustering bacterial cells (Chen et al., 2020; Hu et al., 2023; Tian et al., 2019).

Through the application of state‐of‐the‐art AI methods, we are able to effectively extract information from complex data. As AI research continues, we can only expect easier‐to‐use, better interpretable and more efficient AI methods (Shao et al., 2022).

## CURRENT LIMITATIONS AND FUTURE PERSPECTIVES OF APPLYING AI IN MICROBIOME‐RELATED HEALTHCARE

Despite the progress of using AI in microbiome‐related disease research, few models have found clinical application, likely due to a lack of robustness and generalizability. This can often be attributed to the lack of independent validation or errors during data processing and training. Challenges in training AI models are further aggravated in microbiome research due to enormous variability attributed to a variety of different factors ranging from high impact factors such as geography, diet and medication, via modest effects from host genomics to smaller but variable influences like general lifestyle (Falony et al., 2016; Gupta et al., 2017; Yatsunenko et al., 2012).

Several bottlenecks further hamper the application of AI in microbiome‐related healthcare. Like other biomedical research fields, professionals with expertise in AI technologies and in‐depth domain knowledge are scarce and projections do not expect this to change in the foreseeable future (Glennon et al., 2023). The typical lack of interpretability in AI models is another major bottleneck to translational research. Even if an AI model can successfully solve a given task, it is often very hard to determine on which grounds the model made its decision. While methods like random forests can intrinsically assess how important a given feature is for decision‐making, other ML types require a separate analysis that estimates the impact of individual features (Carrieri et al., 2021; Štrumbelj & Kononenko, 2014). While there are many approaches to tackling the issues of explainable AI, they cannot guarantee a robust explanation of the full model (van der Velden et al., 2022).

An additional frequent issue is data leakage, where data is not properly split into training and test sets, allowing methods to learn shortcuts, leading to overoptimistic reporting and performance failure in real‐world applications (Whalen et al., 2022). This reinforces the need for independent test data, which is often difficult to find (Kim et al., 2017; Papoutsoglou et al., 2023). The acquisition of such independent test data is made significantly easier if a model can make use of unlabeled data, that is, data that has not been reviewed and tagged for a certain purpose. The recently emerging Large Language Models (LLMs) show this capability and allow untrained users such as healthcare professionals or patients to interact with the accumulated knowledge in an intuitive way. LLMs are computational models designed to process and generate natural language, and thus provide a potentially valuable tool to allow intuitive interaction with computational tools. Subject to active research, LLMs have already shown promising results in diagnostic reasoning and clinical conversation in general (Singhal et al., 2023; Tu et al., 2024), while strategies for mitigating the risk of LLMs providing inaccurate information are still sought after (Huang et al., 2023). While further improvements are necessary for LLMs to provide robust medical advice, there have already been pilot projects like GutGPT, that applied LLMs as clinical decision support systems for gastrointestinal bleeding risk (Chan et al., 2023). They can also be applied as a chatbot in patient education, as they can help patients understand and live with their diagnosis (Busch et al., 2024). Although natural language processing is their prime use case, LLMs can also be adopted to decode and understand omics data for different purposes (Liu et al., 2024), such as the prediction of DNA–protein interaction (Zhou et al., 2023), DNA methylation (Jin et al., 2022), protein expression and mRNA degradation (Ren et al., 2024). As training an LLM requires vast computational resources (Chowdhery et al., 2022; Heim, 2022), approaches like transfer‐learning and domain adaptation will be crucial in modifying pre‐trained models towards requirements specific to a country, demographic or phenotype (Karabacak & Margetis, 2023).

A key requirement for the training of effective AI models is access to large‐scale, comprehensive and high‐quality microbiome data. Multiple international efforts for microbiome data collection have been undertaken in the past, like the Human Microbiome Project (Human Microbiome Project Consortium, 2012) and the MetaHIT project (Ehrlich and MetaHIT Consortium, 2011) and paved the way for new projects such as the Million Microbiomes from Humans Project (China National GeneBank (CNGB), n.d.). While such collaborative efforts are instrumental for research, the processing of genetic information and clinical metadata also raises privacy concerns. Microbiome data are considered sensitive information as it is potentially identifying and thus subject to privacy regulations such as the European GDPR (General Data Protection Regulation (GDPR)—Official Legal Text, n.d.). While the GDPR makes concessions for research projects, sharing data between cooperating parties, such as hospitals, is not trivial, particularly in international cooperations. The need for gathering data in one place can be mitigated by federated learning (FL) techniques, where multiple partners can contribute to model training without revealing sensitive data. While FL generally takes more time to execute due to the additional communication overhead, it has been shown that the resulting FL models perform as well as traditional central approaches, even if it is split in a heterogeneous and uneven way (Nasirigerdeh et al., 2022; Zolotareva et al., 2021). Collaborative FL is a promising option for overcoming legal barriers associated with the GDPR and increases the amount of training data, improving performance and generalizability of the resulting models (Asad et al., 2020; Brauneck et al., 2023; Hauschild et al., 2022; Huang et al., 2022). While no microbiome‐specific application of FL has been reported, general‐purpose FL platforms, such as FeatureCloud.ai (Matschinske et al., 2023) or PADME (Jaberansary et al., 2023) could be adapted to microbiome data.

Although the effective implementation of AI in a clinical environment remains challenging, the emergence of new and improved AI methods broadens the scope of possible applications in this environment and has produced many promising avenues to resolve these issues.

## CONCLUSION

In the past, microbiome research mostly focused on studying composition, richness and associations between taxa and disease states as well as microbiome–host interactions. With the increasing availability of diverse and large‐scale datasets across omics layers and the emergence of more sophisticated AI methods, the field is now shifting to investigate the function of microbiomes and interactions within the diverse microbiome communities as well as host–microbiome–drug interactions. Despite these advances, AI has not yet been applied in microbiome‐related healthcare practice. However, AI will likely be key to developing microbiome‐based diagnostics and therapies. In the short‐to‐medium term, diseases with abundant microbiome profiling data or a comparably strong microbiome shift compared to inter‐individual microbiome changes, such as inflammatory bowel disease or colorectal cancer, offer the most realistic prospects of actionable AI‐based healthcare. Still, contemporary methods are not yet suited to fully address the complexity, heterogeneity and diversity of the human microbiome, especially considering its dynamic changes within and across individuals, geographic locations and lifestyles.

With the ever‐increasing need for large amounts of data and computing power, spreading and using open science practices has become increasingly important (‘Open Science’, n.d.; UNESCO, 2021). Initiatives that focus on data collection and availability, such as databases like EMBL‐EBI's MGnify (Richardson et al., 2023) and JGI's IMG/M (Chen et al., 2023) but also larger open science initiatives like European Open Science Cloud (‘EOSC Association’, n.d.) and the Research Data Alliance (‘RDA’, n.d.), build the backbone for enabling widespread access to all resources (data and computational) necessary to effectively engage with AI research. Nonetheless, for AI to pave the way towards personalized medicine, challenges with respect to a lack of robustness and generalization must be overcome. This can be facilitated by improving the amount and quality of training data and its integration across molecular layers and, more importantly, by expanding the use of independent validation data and cross‐regional studies. To facilitate the latter, researchers and clinicians need to account for data privacy regulations and new computing paradigms such as federated learning need to be embraced.

## GLOSSARY

*Artificial intelligence (AI)*: The broader concept of technologies to simulate human intelligence and thus to enable machines to carry out tasks that require a certain amount of intelligence to be solved.

*Curse of dimensionality*: This refers to the situation where many more features are available compared to the number of samples. Each feature adds another degree of freedom to a model. If too few samples are available to robustly train and learn all feature parameters (e.g. coefficients in a regression model), a model is said to suffer from the curse of dimensionality. In this case, problems such as overfitting and a lack of generalization are frequently observed.

*Deep learning (DL)*: A commonly used subset of machine learning techniques that specifically uses neural networks with a large number of layers. This is most frequently applied to tasks with a very large amount of available training data, such as computer vision and natural language processing.

*Explainable AI (XAI)*: Refers to techniques and methods that try to explain the decisions and ‘thought process’ of AI models, helping domain experts to assess the quality of the prediction and to understand the underlying patterns better.

*Feature*: A variable in a dataset such as microbial taxa, protein or metabolite abundance. The more features a dataset has, the higher its dimensionality.

*Generalization*: An ML model is said to generalize if it shows similar performance on independent test data compared to the data it was originally trained and tested on. Independent data ideally comes from a different site.

*Large language model (LLM)*: A deep learning model typically designed to understand and replicate natural human language. The concept has also been applied successfully in biology to represent and study biological sequences.

*Machine learning (ML)*: Machine learning refers to computer algorithms that improve over time through the observation of data. Whereas traditional statistical models are generally aimed at making judgements about a dataset by verifying concrete hypotheses, ML is primarily designed to make predictions or classifications on previously unseen data.

*Microbiome*: The term extends beyond the microorganisms to include their genomes and the surrounding environmental conditions.

*Microbiota*: The collection of microorganisms themselves that reside in a certain defined environment.

*Neural network (NN)*: An ML model that mimics the human brain and consists of layers of interconnected nodes (neurons). The different kinds of NN are often defined by the type of connection between layers, the number of layers or the size of the layers.

*Open science*: Refers to the practice of making scientific research, data, methods and publications freely accessible and transparent to enable reuse and collaboration by anyone.

*Overfitting*: If a model performs excellently on validation data but poorly on test data, this highlights that the model memorized data rather than learning higher level patterns that are needed for a model to generalize to unseen data (see also Generalization).

*Probabilistic ML (PML)*: A subset of ML that uses probabilistic modelling to make predictions and decisions. Key PML methods used in microbiome analysis include hidden Markov models, Bayesian networks and Dirichlet‐multinomial mixture models.

*Random forest (RF)*: A popular ensemble ML method that uses the majority vote of a large number of decision trees. Each decision tree is trained on a random subset of training samples and features.

## AUTHOR CONTRIBUTIONS

**Niklas Probul:** Writing – review and editing; writing – original draft; visualization; conceptualization. **Zihua Huang:** Conceptualization; writing – original draft; writing – review and editing; visualization; funding acquisition. **Christina Caroline Saak:** Conceptualization; writing – original draft; writing – review and editing; supervision; project administration; funding acquisition. **Jan Baumbach:** Conceptualization; funding acquisition; writing – original draft; writing – review and editing; project administration; supervision. **Markus List:** Conceptualization; funding acquisition; writing – original draft; writing – review and editing; project administration; supervision.

## CONFLICT OF INTEREST STATEMENT

The authors declare no conflict of interests.
